# Notes and Notices

**Published:** 1882-05

**Authors:** 


### NOTES AND NOTICES.

Removed.—De Forest Hunt, M. D., has removed to Brooklyn, N. Y., from Grand Rapids, Michigan. Address: 106 St. Mark's Street.

Diphtheria.—Dr. R. R. Gregg, of Buffalo, has a very able paper on "False Membranes of Diphtheria," in the *New York Medical Record* of April 15th. This is the second article the doctor has published on that subject in the same journal.

The L. H. Degree.—After *elevating* Homœopathy to the dignified position of a "therapeutic method," based on the "*rule* similia similibus curantur," the wiseacres, of the London School of Homœopathy, now propose to establish the degree of L. H. (Licentiate in Homœopathy), to advertise the practitioner of the "*method*." Although this degree, it is said, will stamp "the bearer as a man who has *studied* Homœopathy," it appears to us only to mark him as one who knows little or nothing of true Homœopathy. For, we are told, the degree "does not even *bind* him to practice it, *unless he pleases;* and only as far as he *believes* and no further."

Dr. W. M. James, who is in New Mexico for a brief visit, writes us of his success in curing malarial troubles with the single remedy and the minimum dose. The doctor always succeeds by rigidly adhering to Hahnemann's true principles.

Good Pay for Poor Work.—The late President's physicians are to divide among them $85.000 as a slight testimonial from a grateful country for their medical and surgical *skill!* Dr. Bliss gets $25,000 for his successful exhibition of Morphia and Quinia, and his free lectures on "malaria;" Drs. Agnew and Hamilton pocket $15,000 each for stultifying themselves in signing Bliss' bulletins; Mrs. Dr. Edson appropriates $10,000, which is a good fee for a nurse; Dr. Boynton escapes with another $10,000 for his skillful, and often too true, expositions of how Garfield was maltreated.

The Difference.—Allopathy. Visiting physician to interne: "How many deaths since my last visit?" Interne: "Nine, sir." Physician: "Why, I prescribed for ten!" Interne: "Yes, sir; but one would not take the medicine!!"

Homœopathy. Visiting physician to interne: "How many recoveries since my last visit?" Interne: "Nine, sir." Physician: "Why, I prescribed for ten!" Interne: "Yes, sir; but one would not take the medicine!!"

"Jumbo" in Homœopathy.—The newspapers tell us of the wonderful feats of the Elephant "Jumbo," Barnum's recent English acquisition, in disposing of large doses of *Sp. frumenti.* This elephantine feat has only been excelled by a homœopathic physician who announced that he would give his patients (*not himself*) any amount of Quinine, even to a "continent" of it. He exclaims:

> "Let no pent up Utica contract our powers,
> The whole boundless continent [of Peru?] is ours."

